# Soil Acidification in Nutrient-Enriched Soils Reduces the Growth, Nutrient Concentrations, and Nitrogen-Use Efficiencies of *Vachellia sieberiana* (DC.) Kyal. & Boatwr Saplings

**DOI:** 10.3390/plants11243564

**Published:** 2022-12-17

**Authors:** Naledi Zama, Kevin Kirkman, Ntuthuko Mkhize, Michelle Tedder, Anathi Magadlela

**Affiliations:** 1 School of Life Sciences, University of KwaZulu-Natal, Pietermaritzburg Campus, Private Bag X01, Scottsville 3209, South Africa; 2 Agricultural Research Council, Animal Production Institute, Private Bag X02, Irene 0062, South Africa; 3 School of Life Sciences, University of KwaZulu-Natal, Westville Campus, Private Bag X54001, Durban 4000, South Africa

**Keywords:** mesic grassland, nutrient addition, P deficiency, biological nitrogen fixation, liming

## Abstract

Nitrogen (N) and phosphorus (P) nutrient enrichment is important for grasslands. This study aimed to determine how soils enriched with N and P influenced soil concentration correlations and affected the growth kinetics, mineral nutrition, and nitrogen-use efficiencies of *Vachellia sieberiana* grown in a greenhouse experiment. The soils used as the growth substrate were analysed and showed extreme acidity (low soil pH, 3.9). Nitrogen-enriched soils were more acidic than P-enriched soils. Exchangeable acidity was strongly negatively correlated with an increase in soil pH, with soil pH between 3.9 and 4.1 units showing the strongest decline. Plant saplings showed increased root biomass, shoot biomass, total biomass, and plant N and P concentrations when grown in soils with high soil P concentrations. Extreme soil acidification in N-enriched soil was one of the main factors causing P unavailability, decreasing sapling growth. Extreme soil acidification increased concentrations of toxic heavy metals, such as Al which may be alleviated by adding lime to the extremely acidic soils. Research implications suggest that soil pH is an important chemical property of the soil and plays a significant role in legume plant growth. Legume species that are unable to tolerate acidic soils may acquire different strategies for growth and functioning.

## 1. Introduction

In Africa, sustainable plant growth and agricultural practices are threatened by the poor nutrient status of soils [1,2]. Increasing populations worldwide coupled with increased demand for agricultural products are cause for concern, and the need to increase soil fertility in agricultural regions is heightened [3]. South African soils are characterised as having nutrient-poor and acidic conditions [4,5]. The acidic soil pH and low cation-exchange capacity negatively affect the availability of nutrients such as potassium (K^+^) and calcium (Ca^2+^) [6]. In addition, acidic conditions result in the sequestration of certain nutrients like phosphorus (P), causing it to be insoluble through binding with cations [7]. Furthermore, soils with high concentrations of cations including aluminium (Al), iron (Fe) and manganese (Mn) have also been associated with P sequestration and making P unavailable for plant use [8,9]. In sub-Saharan Africa, legumes are important in smallholder farming systems, as they are a source of income and food security [10]. Thus, understanding the impact of soil quality on legume growth and persistence in grasslands is important. Nitrogen (N) and P are important for plant growth and occur in both an organic and inorganic form [11,12,13]. Phosphate also occurs in the energy molecule adenosine triphosphate (ATP), involved in the metabolic activities in plants, and even more in legume plants during biological nitrogen fixation (BNF) [14,15]. A symbiotic relationship may occur between certain legumes and N-fixing bacteria, resulting in BNF [16]. Large amounts of ATP are required as the energy driver for BNF [14]. This is used by microbes in legume plant nodules for metabolic pathways during dinitrogen (N_2_) reduction [17]. Although N is abundantly present in the atmosphere (ca. 78%), soils remain N deficient [18,19]. Legumes can use N in the atmosphere and convert it into usable forms such as nitrate and ammonia through N-fixing microbes housed in the nodules [20]. This emphasizes the importance of BNF in farming and agricultural practices as it reduces the need to apply expensive chemical fertilizers that can be harmful to the environment [18,20]. However, the efficiency of BNF is influenced by certain factors, including P deficiency [17,21], fluctuating pH conditions, temperature and water status [22]. Because P is fixed by cations in acidic soils, thereby making it unavailable to plants [23,24], acidic soil conditions are likely to affect nodulation and N fixation [25,26]. In contrast, large amounts of P support productivity, nodulation, growth, and N fixation in legume plants [27]. 

*Vachellia sieberiana* (DC.) Kyal. & Boatwr (formerly known as *Acacia sieberiana*), commonly known as the paperbark thorn, occurs in nutrient-poor environments and can survive due to its ability to fix atmospheric N [28]. This species encroaches on savannas and mountainous grasslands in the mesic environments of southern Africa [29]. Water availability, water uptake and soil properties are strong determinants of successful tree establishment and encroachment in grasslands [30,31]. Furthermore, growth of *V. sieberiana* saplings is limited by nutrient availability within grassland soils [32]. Research suggests that nutrient-rich soils promote *Vachellia* sapling growth rates when compared to growth in soils of low nutrients [32]. Therefore, it is expected that, soil nutrient status has an extreme effect on legume growth and survival [25,33]. When N was applied to topsoil (0–20 cm), an exponentially negative relationship was observed between N addition rate and N fixation [25,34,35]. It should be noted that nutrient stress can be indirectly caused by changes in soil pH, such as acidity, that suppress nutrient bioavailability, rather than the lack of nutrients themselves [25]. However, when assessing nutrient conditions affecting N fixation, the factors influencing legume growth and those influencing microbe/symbiotic interactions must be distinguished [25]. For instance, water stress and acidity may alter legume root growth, indirectly affecting nodule formation and N fixation [25,36].

Most concerns regarding nutrient addition focus on N and its impact on biodiversity and overall productivity [37,38]. The changing levels of P and K cycles and other nutrients potentially influence the abundance and diversity of legume species in line with resource competition theory [38,39,40,41,42]. Soil nutrient status is important for N-fixing legumes. Owing to their physiological demands, legumes tend to require greater amounts of K, P and other micro-nutrients, including Fe and Ca, than plants that do not fix N [43,44,45,46]. However, altering the natural soil nutrient status by adding nutrients may have synergistic effects [47,48]. Furthermore, limited information is available regarding the long-term nutrient enrichment effects of N, P, and their interaction on soil nutrient status and the growth response and N-use efficiency of *V. sieberiana*. *Vachellia sieberiana* saplings found growing in such ecosystems enriched by nutrients for over 67 years are predicted to show varying growth patterns and mineral nutrition. The Ukulinga Grassland Nutrient Experiment (UGNE) in South Africa is the longest-running nutrient enrichment experiment in Africa [49] and provides a unique opportunity for assessing important ecological and agricultural questions.

The aim of this study was to determine how nutrient-poor soils enriched with N and P influenced soil concentration correlations and affected the growth kinetics, mineral nutrition, and nitrogen-use efficiencies of *Vachellia sieberiana* grown in a greenhouse experiment. We set out to test the following hypotheses:

1. A low soil pH will result in a high exchangeable acidity (EA); thus, there will be negative correlation between the two variables.
2. Soil Al concentration will increase with increasing soil EA and Mn.
3. Soil P concentration will increase with soil Ca concentration, and this phenomenon will be stronger in the P-enriched soils.
4. *Vachellia sieberiana* saplings grown in soils with extremely low pH (below 4) will possess significantly reduced root, stem, leaf, and overall biomass compared to saplings grown in conditions with slightly higher pH (above 4).
5. Sixty-seven years of N and P enrichment will significantly increase both plant N and P concentrations in *V. sieberiana* saplings.
6. *Vachellia sieberiana* saplings grown in N-enriched soils will have significantly reduced nitrogen-use efficiencies compared to those grown in soils not enriched with N.

## 2. Results

### Soil Chemical Properties

**Figure 1:**
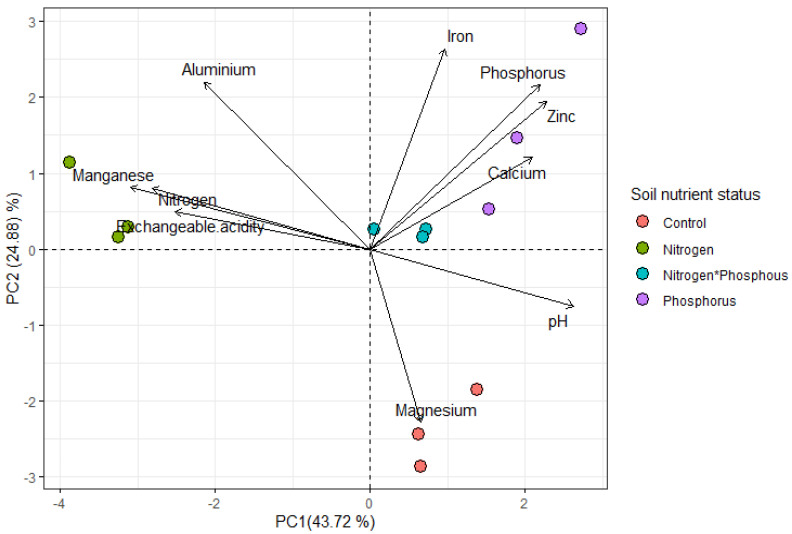
Principal component analysis (PCA) for all standardized soil chemical concentrations under four different soil nutrient status treatments: control (no nutrients), nitrogen (limestone ammonium nitrate), phosphorus only and both nitrogen (limestone ammonium nitrate) and phosphorus. The plot shows the relationship among the soil chemical variables. Positively correlated variables are grouped together, and negatively correlated variables are positioned on the opposite ends from the plot origin. The quality of the variables is assessed by the distance between the variable and the origin. The longer the length of the variables, the greater its level of contribution. Key: control (pink solid circle), N only (green solid circle), N and P (blue solid circle, P only (purple solid circle).

**Figure 2:**
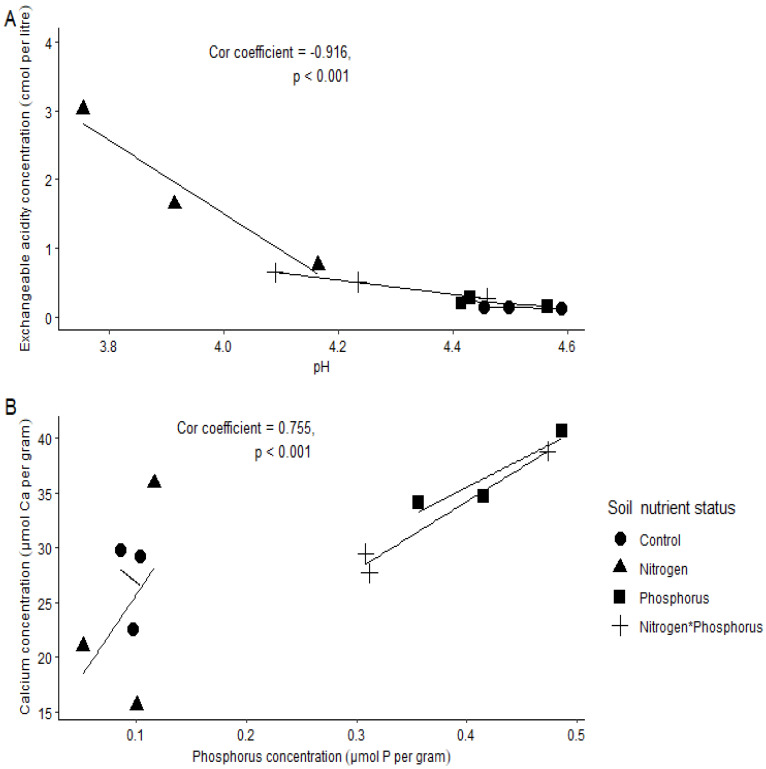
Correlations for soil chemical parameters between (**A**) pH and exchangeable acidity and (**B**) phosphorus and calcium concentrations. Spearman’s correlation coefficient > 0.700 and *p* < 0.05 is shown here. Key: ● = control (no nutrients added); ■ = phosphorus-enriched soils; ▲ = nitrogen-enriched soils and ₊ = nitrogen and phosphorus-enriched soils.

**Figure 3:**
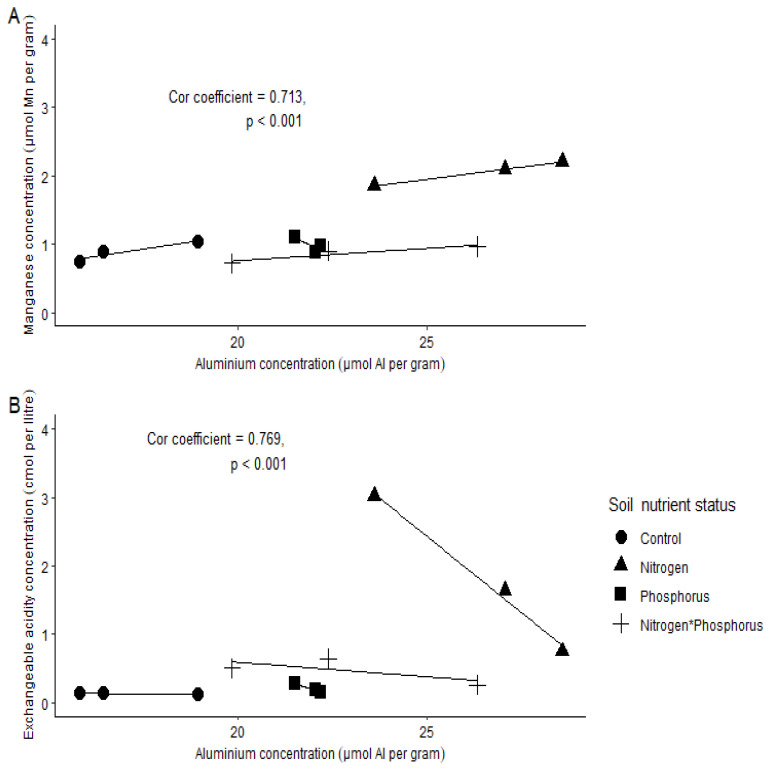
Correlations for soil chemical parameters between (**A**) aluminium and manganese concentrations and (**B**) aluminium and exchangeable acidity. Spearman’s correlation coefficient > 0.700 and *p* < 0.05 is shown here. Key: ● = control (no nutrients added); ■ = phosphorus-enriched soils; ▲ = nitrogen-enriched soils and ₊ = nitrogen and phosphorus-enriched soils.

**Figure 4:**
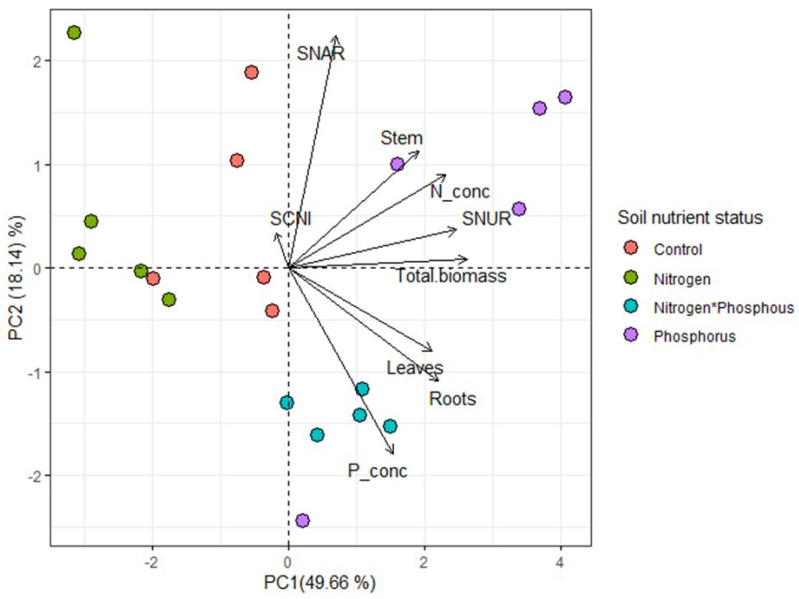
Principal component analysis (PCA) for standardized plant mineral nutrition and growth kinetics trait variables under four different soil nutrient status treatments control (no nutrients), nitrogen (limestone ammonium nitrate), phosphorus only and both nitrogen (limestone ammonium nitrate) and phosphorus. The plot shows the relationship among the plant growth, mineral and nitrogen-use efficiency variables. Positively correlated variables are grouped together, and negatively correlated variables are positioned on the opposite ends from the plot origin. The quality of the variables is assessed by the distance between the variable and the origin. The longer the length of the variables, the greater its level of contribution. Labels: Leaves—leaves biomass (g); Roots—roots biomass (g); stems—stem biomass (g); Total.biomass—overall plant biomass (g); P_conc—plant phosphorus concentration (µmol P. g^−1^); N_conc—plant nitrogen concentration (mmol Ng^−1^); SCNI—standard corrected ^15^N/^14^N; SNAR—specific nitrogen absorption rate (mg Ng^−1^ root dw day^−1^) and SNUR—specific nitrogen use rate (g dw mg^−1^ N day^−1^). Key: control (pink solid circle); N only (green solid circle); N and P (blue solid circle); P only (purple solid circle).

The soil chemical concentrations (µmol g^−1^/Cmol L^−1^) that where highly positively correlated were P and Ca (Figure 1 and Figure 2B), Al and Mn (Figure 1 and Figure 3A), and Al and EA (Figure 1 and Figure 4B). There was a high negative correlation between pH and exchangeable acidity (Ea) (Figure 1 and Figure 2A). High concentrations of Mg (mean ± se, 17.188 ± 0.336) and less acidic conditions (pH = 4.347 ± 0.147) were more associated with control soils (Figure 1 and Table 1). Phosphorus-enriched soils were also associated with slightly higher pH units (when compared to the other treatments) and higher concentrations of Ca, Zn and P (Figure 1 and Table 1). Nitrogen-enriched soils were associated with more acidic soils (low pH units) and high concentrations of Mn, N and EA (Figure 1 and Table 1). Interestingly, soils enriched with both N and P had a more similar soil profile to soils enriched with P only than N only.

We further separated the correlations into their soil nutrient status to assess relationships more closely (Figure 2 and Figure 4). Here, we show only relationships with a correlation coefficient >0.700 and <−0.700 (see Table S1 for addition correlation coefficients). The correlation between pH and EA was negative, but a strikingly steep decline in EA occurred between a soil pH of 3.8 and 4.1. This occurrence was associated with only N-enriched soils (Figure 2A). The correlation between P and Ca revealed that an increase in P concentration was associated with an increase in Ca concentrations, especially in soils enriched either with P only or with both N and P (Figure 2B). However, this was more prominent in the P-enriched soils. The trend for soil Al concentration for the different soil nutrient statuses were as follows: control < NP < P < N. Manganese was slightly increased when Al was higher, this was noticeable in N-enriched soils (Figure 3A). Exchangeable acidity was strongly negatively correlated with increased concentrations of Al in N-enriched soils (Figure 3B).

**Figure 5:**
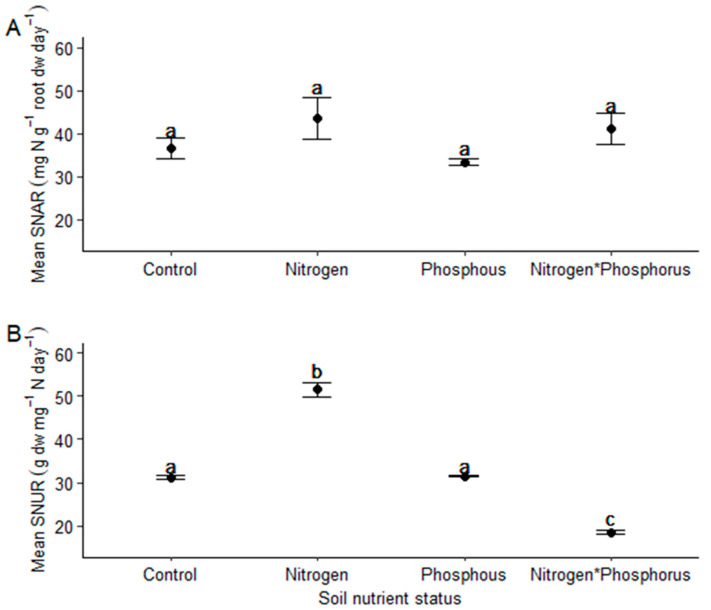
The mean ± standard error (**A**) specific N absorption rate (mg Ng^−1^ root dw day^−1^) and (**B**) specific N use rates (g dw mg^−1^ N day^−1^) of *V. sieberiana* saplings grown in soils with various nutritional statuses: control (no nutrients); nitrogen (limestone ammonium nitrate); phosphorus and both nitrogen (limestone ammonium nitrate) and phosphorus obtained from the Ukulinga Grassland Nutrient Experiment; South Africa. Means ± se are shown here, with different letters indicating significant differences (*p* ≤ 0.05) among treatments, *n* = 5 per treatment.

*Vachellia sieberiana* saplings grown in the different soil nutrient statuses, showed distinct differences in their growth and mineral nutrition (Figure 4). Saplings grown in N only-enriched soils showed significantly less root biomass compared to saplings grown in the other soil conditions (Table 2). Additionally, these saplings also had significantly less leaf biomass and overall biomass compared to saplings grown in both P only enriched and N*P-enriched soils (Table 2). Saplings grown in P-enriched soils had significantly more stem biomass than those grown in N-enriched soils (Table 2). The observation for plant P concentration in saplings grown in the different treatments was as follows; control = N < P = N*P. In contrast, for plant N concentration, it was N < control = P = N*P (Table 2). *Vachellia sieberiana* saplings grown in N-enriched soils had significantly higher SNUR compared to the rest, while saplings grown in N*P-enriched soils had significantly less (Table 2 and Figure 5B).

## 3. Discussion

The long-term effect of nutrient enrichment on the UGNE has resulted in soil chemical variables differentiating based on the single nutrient or nutrient combination treatments applied. We believe that this is one of the main drivers for the differences observed in the growth kinetics, N and P mineral nutrition and N-use demand for *V. sieberiana* plants.

Soils that had been enriched with N, P, and both N and P exhibited a decrease in soil pH by approximately 0.402, 0.108 and 0.084 units on average when compared to soils that had not been enriched with nutrients since 1951. This extreme decrease in soil pH caused by N enrichment is consistent with other long-term studies [50,51]. Estimations indicate that soils are considered to be acidic when top soil pH is below 5.5 and that most of the agricultural land is made up of acidic soils [52]. In this study, soils for all nutrient treatments (including the control) were found to be acidic. However, it is noteworthy to specify that nutrient enrichment of N and P further lowered the soil pH, with N having the greatest decreasing effect. This intensified soil acidification effect is generally associated with numerous conditions that restrict plant growth [53]. Furthermore, soil acidification and leaching reduces the availability of base cations including Ca, Mg, sodium (Na) and K, leading to lower soil fertility and production yield decline [54]. In particular, in this study, N-enriched soils were associated with reduced Ca and Mg concentrations, supporting the notion that leaching of these minerals occurred and could have activated high Al concentrations that would be detrimental to plant growth [55]. When soil pH was below 4.5, the release of high and toxic Al and Mn concentration levels resulted in root damage and decreased overall plant production [56]. Considering the above-mentioned statement, we assume that all the soils used are acidic and potentially have toxic levels of Al and Mn. Surprisingly, this seems to be strongly supported when soils are enriched with N, since the concentrations of Al and Mn were markedly higher. This effect was observed through the significantly lower root biomass in *V. sieberiana* plants grown in N-enriched soils. Our results support hypothesis 1, with a strong relationship being observed between soil pH and exchangeable acidity, where exchangeable acidity decreased with increasing soil pH [51,57]. Exchangeable acidity is defined as the measure of Al^3+^ and H^+^ ions that is retained on the most active constituent of the soil (colloids) [58]. When this is high, and with a resultant low soil pH, the soil conditions and the processes that occur within the soil are affected [59]. We identified a threshold at soil pH = 4.2 following which there appears to be no significant change in exchangeable acidity. The critical changes in decreasing exchangeable acidity occurred between soil pH values of 3.9 and 4.1. However, we observed an increase in EA and Mn with increasing soil Al, which supports hypothesis 2. We associate this with increased soil acidification, especially because under N enrichment, toxic elements such as Al and Mn are released [60,61].

The macronutrients most limiting to photosynthetic production in both aquatic and terrestrial environments is N and P [47,62]. Research on KwaZulu-Natal grasslands has revealed that the soils are acidic, contain cations, and are deficient in P [63]. We further support this notion and maintain that this is true even for soil that has not been enriched with additional nutrients. Consequently, we expected that the measured growth parameters of *V. sieberiana* would be directly influenced by the amount of P available in the soil [64]. The factors that are attributed to low available P include low soil pH (acidic soils) and highly weathered soils, whereby P is absorbed by the presence of soil minerals and P precipitation through Al and Fe [65,66]. In ecosystems with low available P, application of mineral P fertilizer can increase the P content [67]. This is in agreement with our results, in which P concentration in soils enriched with P only and with both N and P were approximately 5.3 and 4.3 times greater than in soils without any nutrient enrichment. Additionally, an increase in soil P concentration was strongly positively associated with an increase in soil Ca concentration, favouring hypothesis 3. We suspect that the increased soil Ca concentration observed here is linked to the calcium-containing superphosphate [Ca(H_2_PO_4_)_2_] P fertilizer [68,69] used in this study. However, chemical fertilizers are used to increase productivity but are also considered to have negative impacts coupled with high costs and further strengthens the motivation for use of organic fertilizer usage instead [70]. Higher soil P availability favours the development and growth of leguminous plants, allowing them to thrive and become dominant within the ecosystems in which they occur [71,72]. In our study, N-enriched soils were associated with extremely low pH and soil P, as well as significantly reduced sapling root, stem, leaf and overall biomass. As a result, we accept hypothesis 4. Additionally, high soil P concentration coupled with high plant biomass of *V. sieberiana* plants occurred in P only- and N and P-enriched soils.

The nutritional status of the soils had a strong effect on both belowground and aboveground biomass of *V. sieberiana* plants. This is supported by soil conditions with higher soil P concentration resulting in greater leaf and root biomass. Ultimately, the same pattern was noticeable for plant P mineral concentration. This was not the case for plant N mineral concentration. Due to these findings, we reject hypothesis 5, indicating that long-term N and P enrichment would increase mineral concentration in *V. sieberiana* saplings. We therefore assume that soil P concentration more strongly positively affects plant P concentration than plant N concentration for *V. sieberiana*. The P requirements for legumes are high, and P deficiencies can impair nodulation and symbiotic N fixation, affecting the growth and respiration of the host [73,74,75]. In this study, plants failed to produce nodules in all soil conditions. A previous study on *Vachellia nilotica* saplings grown in similar N-enriched soil conditions indicated that nodulation did not occur [76]. This occurrence was explained by the low P levels in the soils, considering that P is necessary for regulating energy required for nodule formation and BNF [77]. When nodules do not form, legume species are able to rely on actinomycetes and other Gram-positive bacteria for BNF [78], [79]. We also note that the extreme soil acidity in all soil treatments could have further enhanced the processes that make P unavailable for plant use [80]. We also further emphasize that the P application amount or rate applied at the UGNE over the lifetime of the experiment may not be suitable to support the conditions required for nodule formation in legumes. For example, a 35 percent reduction in nodule numbers for soybean plants occurred when P was oversupplied, and when P was deficient, smaller nodules were present [81]. Hence, we believe that P concentration in the soil is important, and high and low levels can retard structural development and reduce efficient BNF [82,83]. Additionally, N fixation decreases when P is deficient, through an adaptation to low N demand, caused by feedback mechanisms within plants [73]. Therefore, we agree that optimum P concentration is important [84], and thus this should be considered when performing such studies.

*Vachellia sieberiana* saplings grown in N-enriched soils did not have significantly lower nitrogen-use efficiencies when compared to those not enriched with N. Thus, we reject hypothesis 6. Specifically, soil nutrient status did not appear to affect SNAR. This is dissimilar to previous findings on *Vigna unguiculata* saplings grown in soils obtained from soils of varying nutritional status [85]. In this case, a high soil potassium (K) concentration was related to increased SNAR to support increased biomass [85]. We did not consider soil K concentration here, but previous results from the same soils show that K concentrations were 2.531 ± 0.335 (mean ± se) and 4.234 ± 0.681 for the control and P-enriched soils, respectively [16]. However, we believe the main cause for our findings is linked to soil acidification. The soil pH units reported in [16,76] are higher than those reported here, suggesting that more acidic conditions could affect SNAR. In contrast, *V. sieberiana* saplings grown in N-enriched soils under extreme soil acidity and low P concentration showed an increased N use rate (SNUR).

## 4. Materials and Methods

### 4.1. Study Species and Soil Collection Site

The soil samples used to plant the *V. sieberiana* seedlings were collected from the UGNE located at the Ukulinga research farm, University of KwaZulu-Natal, Pietermaritzburg, South Africa (29°24′ E, 30°24′ S). The UGNE is located at an altitude ranging from approximately 838 to 847 m above sea level [86]. The climatic conditions of the site are as follows; mean annual temperature of 18 °C and a mean annual precipitation of approximately 838 mm [87]. The soils are relatively infertile [88]. More specifically, the soil pH differs depending on the source of N in the nutrient application in the plots. A high-level application of ammonium sulphate (ASU) was linked to generally low pH (below 4), whereas high-level limestone ammonium nitrate (LAN) supplemented soils demonstrated slightly higher pH (above 5) [89]. The vegetation is described as a grassland with a few *V. sieberiana* trees accompanied by an understory layer of common grass and forb species [49,89,90].

### 4.2. Experimental Site and Soil Treatment Selection

The UGNE was initiated in 1951 and all plots are mown annually to prevent carry-over plant growth from the previous season [90]. Grazing has been excluded from the study site since its establishment. Originally the experiment was replicated in three blocks, with 96 plots (each 9.0 × 2.7 m) receiving various combinations of N and P. There were two forms of N applied, namely limestone ammonium nitrate (28% N) and ammonium sulphate (21% N) (henceforth LAN and ASU, respectively). For both LAN and ASU, there were four application rates applied annually(0 (control/no nutrients), 7.1, 14.1 and 21.2 gm^−2^). Both N applications were performed twice per year during the months of October and December and were either applied alone or in combination with P ((0 (control/no nutrients) and 33.6 gm^−2^) applied once in October and lime (0 (control/no nutrients) and 225 gm^−2^) applied at five-year intervals [91]. Since superimposing a nutrient cessation experiment on the UGNE in 2018/19, each plot was split into two subplots (4.0 × 2.7 m) separated by a 1 m buffer corridor, with one subplot continuing to receive nutrients, while the other subplot did not. This was then used as the growth substrate for the seedlings to grow in a greenhouse pot trial experiment. We selected soils in the buffer zone from (1) the control plots, (2) LAN-enriched level-2 plots (14.1 g m^−2^), (3) P- enriched plots (33.6 gm^−2^), and (4) plots enriched with both LAN level-2 and P plots, for an interaction effect. This resulted in the four treatments used in this study. We selected LAN over ASU as our N source because it is often used alongside urea in agriculture in South Africa [92], for example, in maize production, and is subject to a range of conversion processes within the soil [92].

In preparation for soil analyses, soil samples were collected from a depth ranging from the surface (0 cm) to 15 cm below the surface. This topsoil region is considered the most active site for fine roots to absorb nutrients [93,94]. For the purposes of this study, we selected only certain plots for soil collection and pooled these together for uniformity. There were three plots per treatment and two soil samples were collected per plot per treatment. We selected Ca, Fe, Mg, Mn, P, N, Zn for our study, since these nutrients are considered important for plant growth and development [95]. Among these, N, P, Ca, and Mg are required in large amounts by plants and thus considered to be macronutrients [95]. Additionally, we included soil pH because it is defined as an important predictor of plant occupancy [96]. Although soil pH is not a primary resource, it does influence nutrient availability and the presence of toxic Al^3+^ cations in soils [97]. Comprehensive soil analyses were performed on the soil samples at the Institute for Commercial Forestry Research located in the Pietermaritzburg Campus of the University of KwaZulu-Natal.

### 4.3. Seed Germination and Growth Conditions

**Figure 6:**
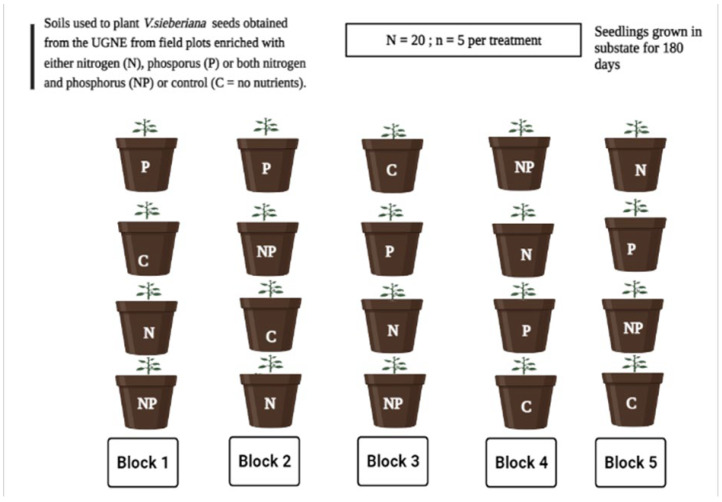
A simple diagram of the layout of treatments in a randomized complete block design in the greenhouse. Key: N = nitrogen, P = phosphorus, NP = nitrogen and phosphorus, and C = control. Sample size *n* = 5.

The pot trial experiment was conducted under ambient conditions in a greenhouse at the Neil Tainton Arboretum, University of KwaZulu-Natal, Pietermaritzburg (30°40′ E, 30°24′ S). Prior to planting, the *V. sieberiana* seeds were soaked overnight in warm water to soften the hard seed coat. Thereafter, the seeds were planted in at a depth of approximately 1–2 cm below the soil surface in pots containing the soils collected from the UGNE. The pot trial experiment was arranged in a randomized complete block design with the four-soil nutrient status treatments (Figure 6). We blocked to account for microenvironmental differences within the greenhouse. Each treatment had a minimum of five replicates, and plants were checked and watered every two days in the morning.

### 4.4. Plant Preparation and Nutrient Analysis

Five plants per treatment were harvested 30 days following seed germination for initial dry weights and N concentrations. Thereafter, final harvesting took place 180 days after the seedlings emerged. During each harvest, the plant material was rinsed with distilled water and separated into roots, leaves, and stems and thereafter oven-dried at 65 °C for four days. Their dry weights were measured and recorded; thereafter plant material was pulverized in a pestle and mortar under laboratory conditions. The ground material was transferred into 2 mL Eppendorf tubes and sent to the Archaeometry Department at the University of Cape Town for C and N isotope analysis and to the Central Analytical Facilities of Stellenbosch University for P analysis.

### 4.5. Calculation of the Specific N Absorption Rate

The initial and final values for total plant *N* were incorporated in the calculation of the specific *N* absorption rate (SNAR). This is described as the net *N* absorption rate by the plant through the roots (mg Ng^−1^ root dw day^−1^):

(1) SNAR =((N_2−N_(1)))/((t_2−t_(1)))  *   ([(〖log〗_(e) R_2−〖log〗_(e) R_1))/((R_2−R_(1))])

where *N* represents the total *N* content present in the plant, *t* represents the period it took the specific plant to grow, and *R* represents the dry root weight [98].

The specific *N* use rate (SNUR) is described as the dry weight gained or acquired by the specific plant during the time of *N* uptake (g dw mg^−1^ N Day-^1^):

(2) SNUR =((W_2−W_(1)))/((t_2−t_(1)))  *   ([(〖log〗_(e) N−〖log〗_(e) N_1))/((N_2−N_(1))])

where *N* represents the total *N* content present in the plant, *t* represents the period it took the specific plant to grow, and *W* represents the plant dry weight [98].

### 4.6. Statistical Analysis

All statistical analyses were performed in R software (version 4.2.1) [99]. To address hypotheses 1, 2 and 3, we conducted separate Principal Components Analyses (PCA) for the soil chemical variables (pH, exchangeable acidity, P, Al, Mn, Zn, Fe, N, Mg, and Ca) and the plant mineral nutrition and growth kinetics traits. We used the “FactoMineR” package in R software [100]; this was supported by the “FactoExtra” package, which is suited for visualization [101]. The benefit of using “FactoMineR” is its ability to transform data to fit a standardized normal distribution. Additionally, we used the “Hmisc” package to assess the correlations between the soil chemical variables by selecting both non-parametric Spearman correlation and parametric Pearson’s correlation as options [102] (Supplementary material, Table S1). To visualize and assess the correlations between the selected soil variables we used Spearman’s rank correlation coefficient as it is a distribution-free statistic [103] that suited the limited soil data samples.

To address hypothesis 3, we carried out our analyses separately for plant traits (N = 20; *n* = five plant seedlings/samples per treatment), plant mineral nutrition and standard corrected ^15^N/^14^N (N = 20; five seedlings/sample per treatment). This was done to meet the assumptions of no multicollinearity. We evaluated the original data with treatment: control (no nutrients), nitrogen, phosphorus, and nitrogen*phosphorus as the grouping independent variable. We tested for the following assumptions of MANOVA: (1) sample size n > number of outcome variables, (2) no univariate outliers, (3) no multivariate outliers, (4) univariate normality, (5) multivariate normality, (6) no multicollinearity, (7) linearity, (8) homogeneity of covariances, and (9) homogeneity of variances. All assumptions of MANOVA were met in both cases. In our assessments, we employed Pillai’s trace as the multivariate test statistic. Additionally, we accepted a statistic significance of *p* < 0.05 in our study. This was followed by Tukey’s HSD to determine the significant difference effects of treatment on the response variables (followed by a Tukey HSD pairwise post hoc comparison test for each response variable). We used the ‘rstatix’ package and the following functions: *identify_outliers*, *shapiro_test*, *cor_test*, *levenes_test* to evaluate four assumptions [104]. To assess linearity, we used the ‘GGally’ package [105]. Lastly, to assess homogeneity of covariances we used the *boxM* function in the ‘heplots’ package [106].

Lastly, to further address hypothesis 3, we performed a separate analysis of variance (ANOVA) for SNAR and SNUR response variables (N = 20, *n* = five individual samples per treatment) to detect significant differences among the independent variables of control (no nutrients), nitrogen, phosphorus, and nitrogen*phosphorus. The assumptions for one-way ANOVA of independence of observations, normal distribution of data for each factor and homogeneity of variances were satisfied. Normality of residuals and homogeneity of variances were assessed using the Shapiro–Wilk test and Levene’s test, respectively. When significant differences were observed (*p* < 0.05), we proceeded with multiple comparisons using the function: *pairwise.wilcox.test* to calculate pairwise comparisons between treatment levels with corrections for multiple testing (*p* < 0.05).

## 5. Conclusions

Our findings show that the soils at the UGNE are acidic, regardless of nutrient enrichment. Therefore, we conclude that applying N at the current rate decreases the soil pH and increases the availability of potential toxic heavy metals. We recommend the addition of lime as a solution to this. It is estimated that to neutralize the acidity from applying 1 kg of N as urea and ammonium sulphate, approximately 1.72 and 5.2 kg of lime would be required, respectively [107].

We acknowledge that under natural conditions, *V. sieberiana* would grow in competition with other plant species and thus recommend competition to be incorporated in future studies. Competition between neighbouring plants can result in trade-offs between defence, production and reproduction [108].

**Figure 7:**
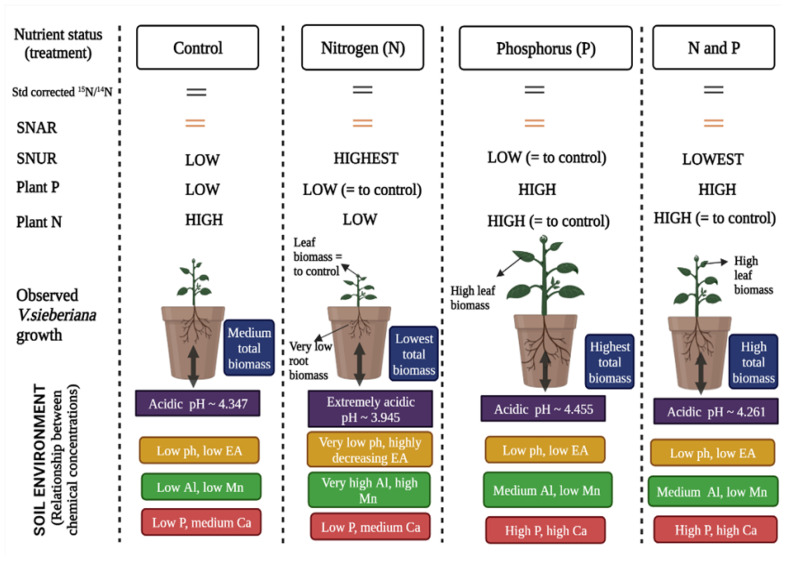
A visual representation of the effects of nitrogen and phosphorus enrichment on soil chemical properties and growth dynamics of *Vachellia sieberiana*. “=” means that the effect of the treatment was non-significant.

*Vachellia sieberiana* is a common tree in the humid grasslands of KwaZulu-Natal, and we concede that further studies incorporating more samples and variables occurring under natural conditions would reinforce our findings (Figure 7). We also recommend that the growth responses of common legumes species with commercial or cultural significance in nutrient-poor soils be investigated.

## Figures and Tables

**Table 1:** The chemical parameters of control soil (no nutrients) and soils enriched with nitrogen (limestone ammonium nitrate), phosphorus and both nitrogen (limestone ammonium nitrate) and phosphorus obtained from the Ukulinga Grassland Nutrient Experiment, South Africa.

Soil Parameter	Control	Nitrogen	Phosphorus	Nitrogen * Phosphorus
pH (KCl)	4.347 ± 0.147	3.945 ± 0.119	4.455 ± 0.089	4.261 ± 0.107
Exchangeable acidity	0.998 ± 0.809	1.799 ± 0.659	0.218 ± 0.031	0.471 ±0.112
Aluminium	17.071 ± 0.954	26.409 ± 409	21.914 ± 0.207	22.854 ± 1.883
Calcium	27.224 ± 2.327	24.156 ± 6.071	36.519 ± 2.101	32.003 ± 3.445
Iron	1.328 ± 0.059	1.586 ± 0.104	2.064 ± 0.322	1.444 ± 0.148
Magnesium	17.188 ± 0.336	11.838 ± 2.824	12.025 ± 0.999	10.446 ± 1.361
Manganese	0.892 ± 0.084	2.051 ± 0.105	0.988 ± 0.062	0.863 ± 0.074
Phosphorus	0.084 ± 0.012	0.090 ± 0.020	0.451 ± 0.042	0.364 ± 0.054
Nitrogen	0.002 ± 0.522	0.029 ± 0.002	0.024 ± 0.001	0.023 ± 0.001
Zinc	0.017 ± 0.006	0.013 ± 0.001	0.048 ± 0.003	0.017 ± 0.005

Each value is a mean ± standard error of three replicates.

**Table 2:** *Vachellia sieberiana* biomass and mineral nutrition grown in control soils (no nutrients) and soils enriched with nitrogen (limestone ammonium nitrate), phosphorus and both nitrogen (limestone ammonium nitrate) and phosphorus obtained from the Ukulinga Grassland Nutrient Experiment, South Africa. Different letters indicate significant differences among treatments per parameter, at *p* ≤ 0.05.

Plant Traits				
Parameter	Control	Nitrogen	Phosphorus	Nitrogen * Phosphorus
Roots (g)	4.379 ± 0.623 ^b^	2.269 ± 0.464 ^a^	5.828 ± 0.587 ^b^	5.815 ± 0.368 ^b^
Leaves (g)	0.769 ± 0.070 ^ab^	0.696 ± 0.079 ^a^	1.211 ± 0.125 ^bc^	1.282 ± 0.176 ^c^
Stems (g)	2.972 ± 0.866 ^ab^	2.367 ± 0.578 ^a^	5.909 ±1.400 ^b^	2.954 ± 0.240 ^ab^
Total biomass (g)	8.119 ± 0.604 ^ab^	5.331 ± 1.034 ^a^	12.949 ± 1.482 ^b^	10.051 ± 0.553 ^b^
**Plant mineral Nutrition**				
Parameter	Control	Nitrogen	Phosphorus	Nitrogen * Phosphorus
Total plant P (µmol P. g^−1^)	25.319 ± 2.779 ^a^	26.699 ± 1.139 ^a^	37.193 ± 2.433 ^b^	41.306 ± 1.963 ^b^
Total plant N (mmol Ng^−1^)	48.017 ± 3.046 ^b^	31.137 ± 2.697 ^a^	56.851 ± 6.259 ^b^	48.171 ± 0.972 ^b^
Standard corrected ^15^N/^14^N	6.021 ± 0.886 ^a^	4.562 ± 0.505 ^a^	3.896 ± 1.142 ^a^	5.405 ± 0.283 ^a^

Each value is a mean ± standard error of five replicates. Values with the same letter superscript within a row under the same parameters are not significantly different at *p* ≤ 0.05.

## Data Availability

All raw data and R scripts will be available upon request from Anathi Magadlela, email: MagadlelaA@ukzn.ac.za.

## Supplementary Materials

The following supporting information can be downloaded at: https://www.mdpi.com/article/10.3390/plants11243564/s1, Table S1. Pearson’s and Spearman’s correlation for the soil chemical parameters. Distribution of all soil variables were not performed; thus, we show both parametric and non-parametric statistical procedures.
