# Supplementary material for: Soil Acidification in Nutrient-Enriched Soils Reduces the Growth, Nutrient Concentrations, and Nitrogen-Use Efficiencies of Vachellia sieberiana (DC.) Kyal. & Boatwr Saplings
Source: Plants (Basel). 2022 Dec 17;11(24):3564. doi: 10.3390/plants11243564 (PMC9781205; doi:10.3390/plants11243564)
Supplement: Supplementary file 1 [file plants-11-03564-s001.zip › plants-2059904-supplementary.pdf]

Supplementary material

Table S1. Pearson's and Spearman's correlation for the soil chemical parameters. Distribution of all soil variables were not performed; thus we show both parametric and non-parametric statistical procedures

**Soil variable 1**	**Soil variable 2**	**Pearson's correlation**	**Spearman's correlation**
Aluminium	Calcium	-0.228	-0.070
Aluminium	Magnesium	-0.676	-0.545
Calcium	Magnesium	0.271	0.350
Aluminium	Manganese	0.738	0.713
Calcium	Manganese	-0.489	-0.126
Magnesium	Manganese	-0.223	-0.049
Aluminium	Zinc	-0.116	-0.182
Calcium	Zinc	0.596	0.594
Magnesium	Zinc	-0.090	0.077
Manganese	Zinc	-0.324	-0.021
Aluminium	pH	-0.506	-0.573
Calcium	pH	0.095	0.021
Magnesium	pH	0.102	0.196
Manganese	pH	-0.684	-0.329
Zinc	pH	0.402	0.133
Aluminium	Exchangeable acidity	0.420	0.769
Calcium	Exchangeable acidity	-0.006	-0.056
Magnesium	Exchangeable acidity	0.119	-0.357
Manganese	Exchangeable acidity	0.687	0.524
Zinc	Exchangeable acidity	-0.346	-0.133
pH	Exchangeable acidity	-0.909	-0.916
Aluminium	Nitrogen	0.586	0.622
Calcium	Nitrogen	-0.456	-0.378

Magnesium	Nitrogen	-0.288	-0.266
Manganese	Nitrogen	0.802	0.483
Zinc	Nitrogen	-0.417	-0.545
pH	Nitrogen	-0.559	-0.448
Exchangeable acidity	Nitrogen	0.547	0.587
Aluminium	Phosphorus	0.036	0.014
Calcium	Phosphorus	0.730	0.755
Magnesium	Phosphorus	-0.354	-0.231
Manganese	Phosphorus	-0.523	-0.329
Zinc	Phosphorus	0.658	0.538
pH	Phosphorus	0.230	-0.028
Exchangeable acidity	Phosphorus	-0.350	0.049
Nitrogen	Phosphorus	-0.387	-0.301
Aluminium	Iron	0.188	0.280
Calcium	Iron	0.386	0.203
Magnesium	Iron	-0.324	-0.462
Manganese	Iron	-0.022	0.196
Zinc	Iron	0.708	0.476
pH	Iron	0.117	-0.007
Exchangeable acidity	Iron	-0.060	0.147
Nitrogen	Iron	0.040	0.000
Phosphorus	Iron	0.478	0.406
